# Cell Membrane-Interrupting Antimicrobial Peptides from *Isatis indigotica* Fortune Isolated by a *Bacillus subtilis* Expression System

**DOI:** 10.3390/biom10010030

**Published:** 2019-12-24

**Authors:** Jia Wu, Hafiz Muhammad Khalid Abbas, Jiale Li, Yuan Yuan, Yunjun Liu, Guoying Wang, Wubei Dong

**Affiliations:** 1 Department of Plant Pathology, College of Plant Science and Technology and the Key Lab of Crop Disease Monitoring & Safety Control in Hubei Province, Huazhong Agricultural University, Wuhan 430070, Hubei Province, China; wj123@webmail.hzau.edu.cn (J.W.); ljl0410@webmail.hzau.edu.cn (J.L.); 2 Vegetable Research Institute, Guangdong Academy of Agricultural Sciences, Guangzhou 510640, China; khalid_mpp@hotmail.com; 3 Guangdong Key Laboratory for New Technology Research of Vegetables, Guangdong Academy of Agricultural Sciences, Guangzhou 510640, China; 4 National Resource Center for Chinese Materia Medica, China Academy of Chinese Medical Sciences, Beijing 100700, China; y_yuan0732@163.com; 5 Institute of Crop Science, Chinese Academy of Agricultural Sciences, South Street of Zhongguancun 12, Beijing 100081, China; liuyunjun@caas.cn (Y.L.); wangguoying@caas.cn (G.W.)

**Keywords:** antimicrobial peptide, plant resistance gene, membrane-interrupting peptides, gene expression system, *Bacillus subtilis*, *Isatis indigotica*

## Abstract

The situation of drug resistance has become more complicated due to the scarcity of plant resistance genes, and overcoming this challenge is imperative. *Isatis indigotica* has been used for the treatment of wounds, viral infections, and inflammation for centuries. Antimicrobial peptides (AMPs) are found in all classes of life ranging from prokaryotes to eukaryotes. To identify AMPs, *I. indigotica* was explored using a novel, sensitive, and high-throughput *Bacillus subtilis* screening system. We found that IiR515 and IiR915 exhibited significant antimicrobial activities against a variety of bacterial (*Xanthomonas oryzae*, *Ralstonia solanacearum*, *Clavibacter michiganensis*, and *C. fangii*) and fungal (*Phytophthora capsici* and *Botrytis cinerea*) pathogens. Scanning electron microscope and cytometric analysis revealed the possible mechanism of these peptides, which was to target and disrupt the bacterial cell membrane. This model was also supported by membrane fluidity and electrical potential analyses. Hemolytic activity assays revealed that these peptides may act as a potential source for clinical medicine development. In conclusion, the plant-derived novel AMPs IiR515 and IiR915 are effective biocontrol agents and can be used as raw materials in the drug discovery field.

## 1. Introduction

Drug resistance in pathogenic microbes is an emerging challenge in crop production and human health care [1,2]. Multiresistant bacteria have many strategies that threaten the health of animals and plants [3], such as beta-lactamases that are enzymes synthesized by bacteria to break host resistance [4]. This situation has been provoked by the recent attention paid to identifying new antimicrobial genes against emerging bacterial resistance [5]. Therefore, scientists have been trying to discover novel biocontrol agents to overcome this problem. Antimicrobial peptides (AMPs) have received a considerable amount of attention because of their significant activity against fungi, bacteria, parasites, viruses, inflammation, and tumor cells [6,7]. It is generally believed that AMPs are short peptides (20–50 amino acids) with low molecular weight and mostly linear cationic α-helices [8], with effective activity against a broad spectrum of pathogens. AMPs can be isolated from a variety of organisms such as bacteria, fungi, insects, plants, animals, and humans [9,10]. As a new class of antibiotic with low tendency to induce resistance, high antimicrobial activity, and good selectivity, AMPs have the potential to replace some traditional antibiotics in the future [11]. The Gram-positive bacterial cell envelope is composed of an outer cell wall (a thick peptidoglycan layer and a polysaccharide coat) and an inner cytoplasmic membrane. To achieve their bactericidal activities, AMPs interact with the cell wall or cytomembrane, resulting in membrane interruption and cell lysis [12]. In most of these cases, AMPs are reported to cause disruptions in cell wall or cell membrane integrity, perforation, deformation, and increased water ion and molecular flow across the membrane, which ultimately causes microbial death [13].

In biotechnology, *Bacillus subtilis* has been considered an effective tool to study high-level expression of foreign proteins [14]. Additionally, it is a generally regarded as safe (GRAS) organism that does not produce endotoxins [15]. Because of its relatively simple cell structure, high growth rate, short fermentation time, and high capacity to secrete proteins directly into the extracellular medium [16], it has long been successfully used for the expression of many protein products, including some industrial enzymes (proteases, lipases, and amylases) [17,18]. 

*Isatis indigotica* belongs to the Brassicaceae family and is a biennial herb that has been used as a traditional medicine to cure wounds in Europe and China for centuries [19]. Different compounds isolated from *I. indigotica* leaves have exerted anti-inflammatory and anti-allergic activities [20]. Extracts of *I. indigotica* hairy root cultures showed antioxidant activities [21]. Alkaloids isolated from *I. indigotica* exhibited inhibitory activities against two different types of ureases (Jack bean and *Bacillus pasteurii* ureases) and significant antifungal activity against *Aspergillus niger*, *Candida albicans*, *Trichophyton schoenleinii*, *T. simii*, and *Macrophomina phaseolina* [22]. 

To date, most of the research related to AMPs has been focused on extraction, separation, purification, and synthesis of AMPs, as well as some exploration of resistance mechanisms [23]. For the isolation of candidate AMPs, there are two main methods. One method is based on stepwise separation and detection of proteins or polypeptides after isolation from organisms directly [24]. Because the number of AMPs in microbes is usually limited, extensive losses can occur during the isolation and purification procedures. The second method is to synthesize AMPs artificially. This method can improve AMP efficacy and range of performance.

Two factors contribute to a bottleneck in plant resistance breeding—the scarcity of plant resistance genes and the fact that resistance genes are easily overcome by pathogens. To overcome the scarcity of plant resistance genes, we established an antimicrobial gene isolation method using a *B. subtilis* expression system [25]. To avoid the propensity for resistance genes to be overcome by pathogens, we designed a model in which pathogen-independent, nonself-recognition triggered, and heterosis-based fresh resistance can be generated in F1 hybrids [26]. To the best of our knowledge, *I. indigotica* has been less well documented for its antimicrobial potential. In the current study, novel AMPs were identified from *I. indigotica* using the *B. subtilis* expression system, and further studies were performed to explore their antibacterial and antifungal activities. To determine the basis of the host–pathogen interaction, the potential for these AMPs to control plant diseases and their mechanisms were also investigated in this study.

## 2. Results

### 2.1. Candidate Genes from an Isatis indigotica cDNA Library Exhibited Antimicrobial Potential

**Figure 1:**
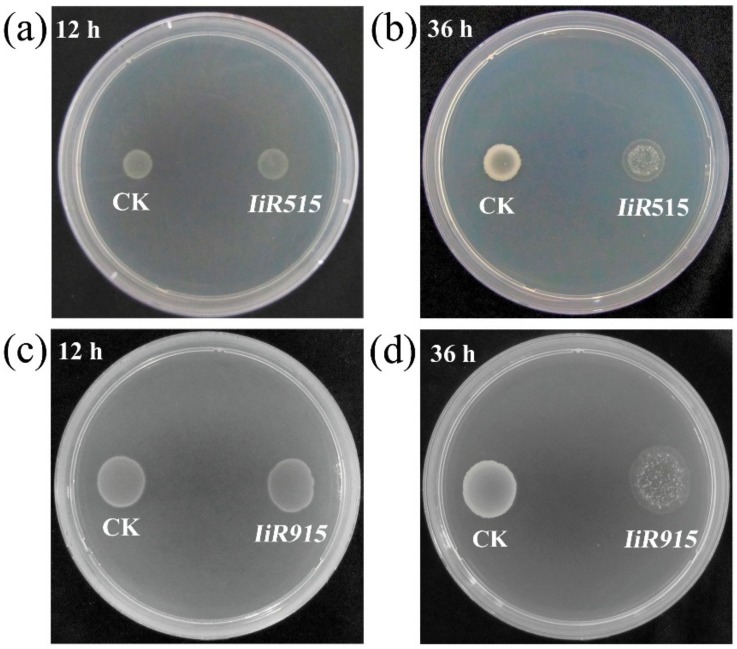
Damaging effect of foreign proteins on host *Bacillus subtilis* cells. The engineered *B. subtilis* strains harboring *IiR515*, *IiR915*, and empty vector were separately spotted onto Luria-Bertani (LB) plates and incubated at 37 °C. (**a**) *IiR515* after 12 h of incubation and (**b**) after 36 h of incubation; (**c**) *IiR915* after 12 h of incubation and (**d**) after 36 h of incubation. The empty vector-transformed *B. subtilis* strain (WB800-e) was used as a control. Spots on the right represent the test clones with damaging effects and spots on the left represent the control.

In an attempt to identify AMPs from *I. indigotica*, a cDNA library was constructed from purified mRNA using the pBE-S vector and *B. subtilis* expression system (Figure S1). We assumed that the expression and secretion of candidate genes from the cDNA library in *B. subtilis* cells would reveal the killing or damaging effects of the expressed products in their virulence against host cells. Based on this assumption, cells with abnormal growth were identified and their inserts were considered to be potential antimicrobial genes (Figure 1). 

The lengths of the inserts were analyzed by polymerase chain reaction (PCR) (Figure S2), and the quality of the cDNA library was determined based on the primary library titer and recombination rate, which were 5.6 × 10^6^ CFU/mL (Colony Forming Units per milliliter) and 90.27%, respectively. During initial screening, a total of 2.20% of clones (45/2039) demonstrated killing effects on *B. subtilis*, and among them, 15 clones showed strong antimicrobial activities. These results suggested that the inserted fragments in those 15 clones coded for products with the potential to inhibit or kill microorganisms (Table S2). Based on this screening, *IiR515* and *IiR915* were selected for further study.

### 2.2. Candidate Antimicrobial Peptides Destroyed the Cell Membrane of B. subtilis

**Figure 2:**
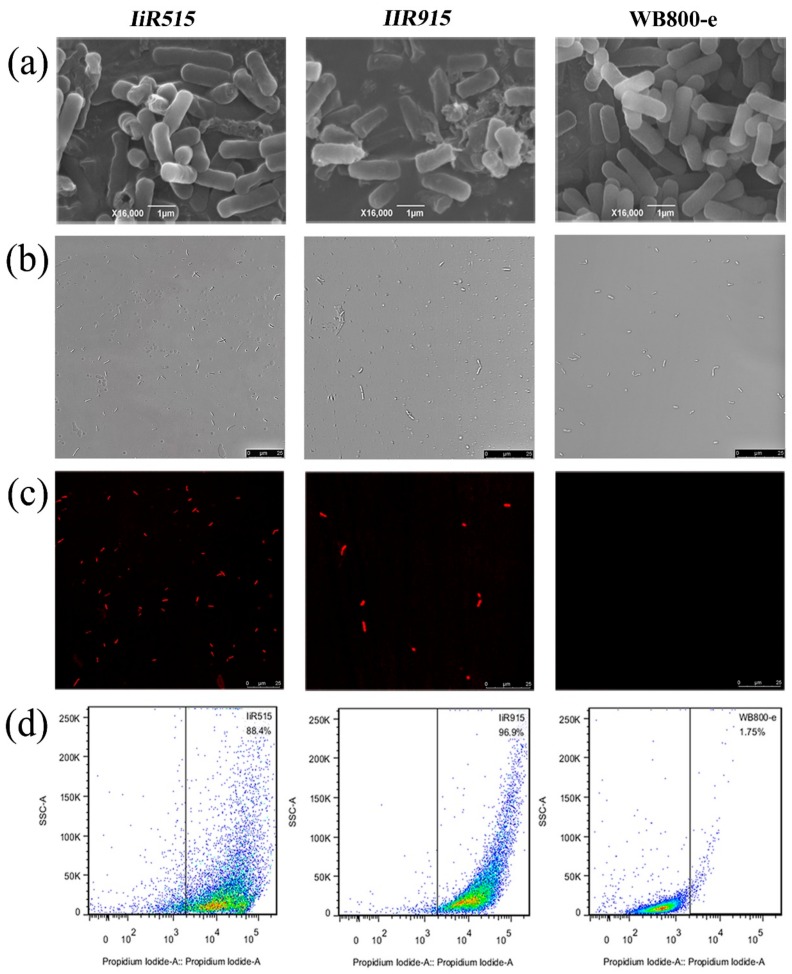
Cell membrane disruptions of transformed *B. subtilis* cells. *Bacillus* pellets were fixed with glutaraldehyde after washing with PBS buffer (phosphate buffer saline). Finally, the samples were lyophilized and then gold coated. (**a**) Cellular disruption of *IiR515*, *IiR915*, and WB800-e (empty vector-transformed *B. subtilis* WB800) under the scanning electron microscope. Test bacteria were washed and resuspended in PBS buffer at 1 × 10^9^ CFU/mL and stained with PI (40 mg/mL). Confocal images under (**b**) ordinary light and (**c**) fluorescence. (**d**) Percentages of fluorescent events (relative value of PI staining) in gate shown in the region on the right. The *x*-axis shows the relative fluorescence intensity and the *y*-axis shows the side scatter light.

To determine the damaging effects of the functional peptides on the cytomembrane, scanning electron microscopy (SEM), confocal microscopy, and cytometric analysis were used to observe the cellular surface morphology and integrity (Figure 2). From the SEM results, the control *B. subtilis* WB800-e strain showed normal and intact cell morphology. By contrast, membrane damage, such as membrane holes, deformation, and lysis, was observed in cells transformed with the *IiR515* and *IiR915* genes (Figure 2a). Cell staining was observed by confocal imaging. Under an ordinary optical microscope, rod-shaped bacterial cells were observed for both the control and transformed strains (Figure 2b). However, images under a fluorescence microscope were different; red fluorescence was not observed for *B. subtilis* WB800-e (control), whereas obvious red rod-shaped *IiR515-* and *IiR915*-transformed bacteria were observed (Figure 2c). Propidium iodide (PI) uptake increases with an increase in cell membrane permeability. Dot plots showed that the *B. subtilis* WB800-e strain had minimal PI staining (approximately 1.75%). Conversely, the PI fluorescent signal for the transgenic strains was much stronger, approximately 88.4% for *IiR515* and 96.9% for *IiR915* (Figure 2d). 

The *y*-axis shows the intensity of scattered light, which also increased with increased PI fluorescence signal (*x*-axis). All of these results showed that the intact cell membrane of wild-type *Bacillus* rejected the infiltration of PI. By contrast, PI infiltration into the *IiR515-* and *IiR915*-transformed cells occurred because of their incomplete cell membranes and PI bound to their exposed nucleic acids, resulting in the observation of red fluorescence. Our results revealed that the cell membrane was significantly destroyed, ultimately leading to cell death.

### 2.3. Candidate Antimicrobialpeptides Altered the Fluidity and Electrical Potential of the Bacillus subtilis Cytoplasmic Membrane 

**Figure 3:**
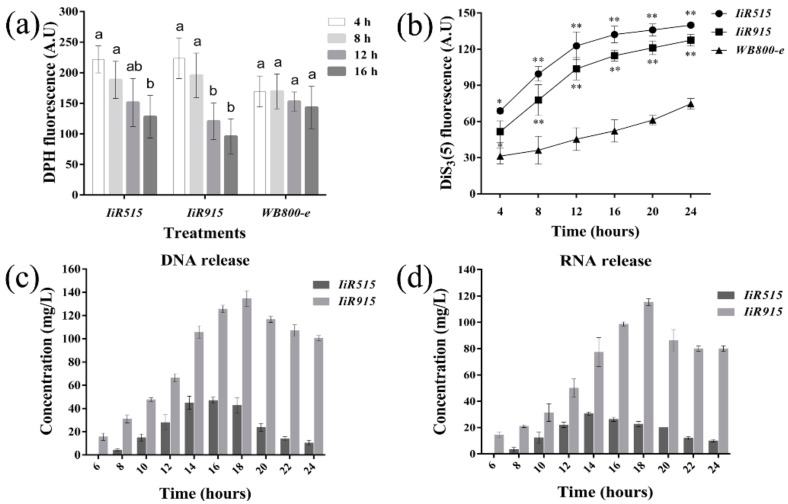
Fluorescence intensity and nucleic acid release of *B. subtilis* cells. The fluidity and electrical potential of the cytoplasmic membrane of *B. subtilis* were measured. Excitation and emission wavelengths for (**a**) The DPH were 365 nm and 425 nm and for (**b**) DiSC_3_(5) were 622 nm and 670 nm, respectively. The WB800-e strain was used as a control. The detection of released DNA and RNA was also performed. The empty vector was used as a blank control. (**c**) The concentration of DNA and (**d**) RNA in shaking media at different time intervals. Data are the mean values from three individual experiments. Vertical bars represent the SD (Standard deviation). For significance analysis, *t*-tests were performed; * *p* < 0.05, ** *p* < 0.01.

For the further analysis of the integrity of the cytoplasmic membrane, the fluidity and electrical potential (ΔΨ) of the cytoplasmic membrane were monitored. The DPH (1.6-diphenyl-1,3,5-hexatriene) fluorescent probe was used for orientation, predominantly parallel to the fatty acid chains, and reflected the fluidity of the core of the cytoplasmic membrane, while 3,3’-dipropylthiadicarbocyanine iodide (DiSC_3_(5)) was used to detect perturbations of the electrical potential gradients across the cytoplasmic membrane. Our results showed that for *IiR515-* and *IiR915*-transformed *Bacillus* cells, the fluidity of the plasma membrane core was significantly reduced as time increased, but changes were not significant for the control WB800-e strain (Figure 3a). The DiSC_3_(5) fluorescent probe was used to detect perturbations in electrical potential gradients across the cytoplasmic membrane, and we observed that changes in the electrical potentials of *IiR515-* and *IiR915*-transformed *Bacillus* cells were significantly higher than the control cells (Figure 3b).

### 2.4. The Detection of DNA/RNA in Shaking Media Indicated Cell Membrane Breakage

We assumed that if the membranes of transformed strains were damaged, then some of their components, such as nucleic acids (DNA and RNA), would leach out into the media. To prove this assumption, the total amount of released DNA and RNA was measured using an Eppendorf Bio Photometer. The results supported our hypothesis that *IiR515* and *IiR915* made pores in the cell membrane to release nucleic acids out into the media (Figure 3c). The highest amount of DNA was observed for *IiR515* at the 16 h time point, whereas the highest levels for *IiR915* were observed at the 18 h time point. A significant amount of RNA was also observed in the media at other time intervals (Figure 3d).

### 2.5. Extracellular Peptides of IiR515 and IiR915 Exhibited Antimicrobial Activities

**Figure 4:**
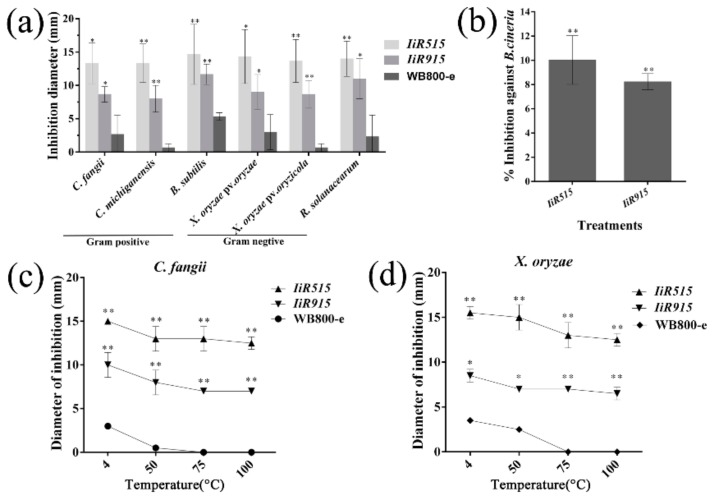
Analysis of potential antimicrobial peptides (AMPs) against pathogens. For antimicrobial activity assays, the *B. subtilis* WB800-e strain was used as a control. (**a**) Inhibition of Gram-positive and Gram-negative bacteria in response to *IiR515* and *IiR915* compared to WB800-e. (**b**) Percent inhibition of IiR515 and IiR915 against *Botrytis cinerea* compared to WB800-e. Temperature curves for (**c**) *Clavibacter fangii* and (**d**) *Xanthomonas* oryzae. Data are the mean values from three individual experiments. Vertical bars represent the SD. For significance analysis, *t*-tests were performed; * *p* < 0.05, ** *p* < 0.01.

Extracellular peptides of all candidate strains were extracted using the ammonium sulfate precipitation method and tested for their antimicrobial activities. The results revealed that the IiR515 and IiR915 peptides exhibited significant levels of inhibition against Gram-positive and Gram-negative bacteria as well as fungi compared to controls (Figure 4a,b and Figure S3). Thermal stability assays revealed that both peptides acted as stable antimicrobial agents against Gram-positive and Gram-negative bacteria at temperatures ranging from 4 to 100 °C (Figure 4c, d). After purification, the minimum inhibitory concentrations (MICs) of IiR515 and IiR915 were evaluated using the critical dilution method against Gram-positive (*Clavibacter fangii* and *C. michiganensis*) and Gram-negative (*Xanthomonas oryzae* and *Ralstonia solanacearum*) bacteria. Results indicated that the MICs of both peptides were less than 100 µg/mL (Table S4).

### 2.6. Western Blots Revealed the Expression and Size of the IiR515 and IiR915 Peptides

**Figure 5:**
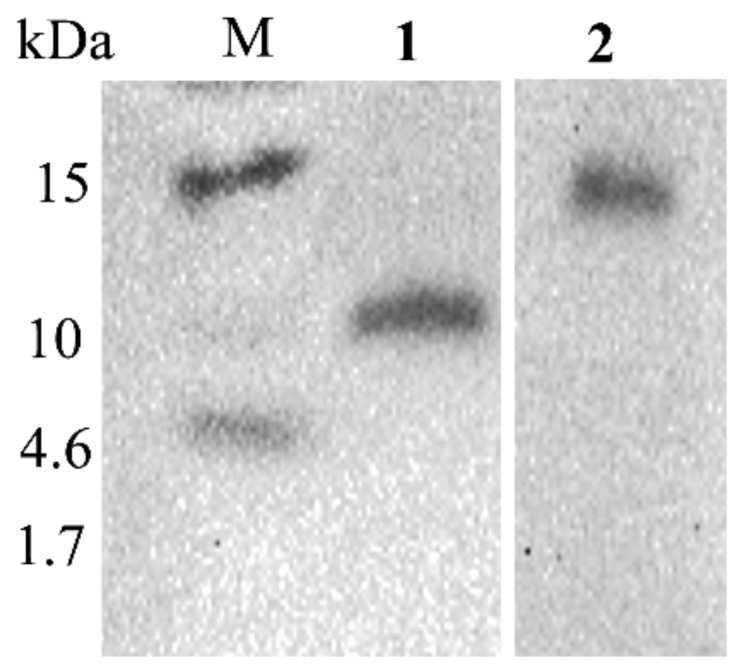
Western blot analysis of fusion peptides.

His-tag fusion IiR515 and IiR915 peptides were expressed in B. subtilis WB800 cells. Extracellular peptides were purified using a nickel column and tested for their expression by Western blot. The molecular weights of IiR515 (14 kDa) and IiR915 (10 kDa) were observed from a PVDF (Polyvinylidene fluoride) membrane using autoradiography (Figure 5), and we found that the apparent sizes were larger than their predicted sizes. From previous reports, it has been observed that the relatively higher isoelectric point, the strong basic charge of the peptides, and the His-tag fusion exert a significant impact on peptide movement in sodium dodecyl sulphate polyacrylamide gel electrophoresis (SDS-PAGE) gels [27]. These results prove that our peptides of interest accumulate in growth media as expected.

Autoradiography of IiR515 and IiR915 fusion peptides is shown in Figure 5. Lane M indicates the color prestaining ultra-low molecular weight (1.7-40 kDa) marker. Lane 1 indicates the molecular weight of the full-length IiR915 peptide (10 kDa). Lane 2 indicates the molecular weight of the full-length IiR515 peptide (14 kDa).

### 2.7. IiR515 and IiR915 Prevented Phytophthora capsici and Botrytis cinerea Infection on Detached Leaves of Nicotiana benthamiana 

**Figure 6:**
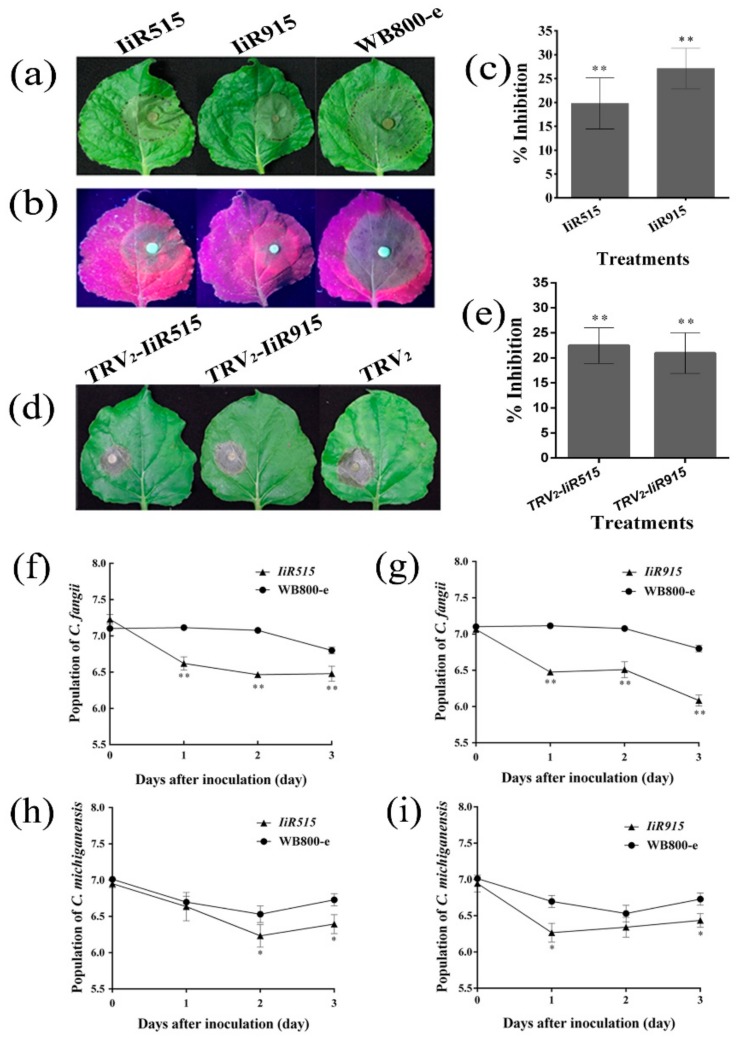
Percent inhibition of *Phytophthora capsici* and *Botrytis cinerea* on *Nicotiana benthamiana* leaves. Images of *P. capsici* disease inhibition (**a**) under normal light and (**b**) ultraviolet light (365 nm). (**c**) Percent inhibition compared to the control. (**d**) Images of *B. cinerea* inhibition and (**e**) percent inhibition compared with the control. The *y*-axis shows the logarithmic values of the bacterial population (log CFU/10 g of soil). The inhibitory potential of (**f**) the *IiR515* strain and (**g**) the *IiR915* strain against *C. fangii*. Inhibitory potential of (**h**) the *IiR515* strain and (**i**) the *IiR915* strain against *C. michiganensis* compared to the control WB800-e strain. Data are the mean values from three individual experiments. Vertical bars represent the SD. For significance analysis, *t*-tests were performed; * *p* < 0.05, ** *p* < 0.01.

Sensitivity assays using purified IiR515 and IiR915 were performed on detached leaves of *N. benthamiana* under controlled conditions. At 48 h post-inoculation (hpi), images were taken under normal and ultraviolet (UV) light (Figure 6a,b). Results indicated a significant percent inhibition of *P. capsici* compared to the control (Figure 6c). Leaves were infiltrated with *TRV_2_-IiR515* and *TRV_2_-IiR915*, and then disks with *B. cinerea* were used to inoculate on the other side of the infiltrated leaves at 48 hpi. Images were taken at 48 h after fungal inoculation (Figure 6d). The data revealed that IiR515 and IiR915 have the ability to restrict the growth of fungal pathogens (Figure 6e). 

### 2.8. IiR515- and IiR915-Transformed Bacillus subtilis Inhibited the Growth of Soil-Borne Pathogens 

Anti-soil-borne bacterial assays were applied in simulated soil environments. Plant pathogens were applied to soil treated with transformed *B. subtilis*. The results showed restricted growth of pathogens in soil treated with the *IiR515* and *IiR915* strains (Figure 6). We observed that inoculation with the *IiR515* strain had a maximum inhibitory effect on *C. fangii* and *C. michiganensis* growth on the 2nd and 3rd day, respectively (Figure 6f,h), whereas in the case of the *IiR915* strain, maximum restriction of both *C. fangii* and *C. michiganensis* was observed on the 1st day (Figure 6g,i).

### 2.9. IiR515 and IiR915 Peptides Showed No Significant Hemolytic Activity

Hemolytic activity assays were performed against sheep blood cells. We found that the hemolysis rates of the IiR515 and IiR915 purified peptides at the extremely high concentration of 1000 mg/L were 5.3% and 7.5%, respectively (Table S5). According to our results, no significant hemolytic activity was observed against sheep blood cells. We can likely conclude that these peptides are relatively safe for mammalian cells.

## 3. Materials and Methods

### 3.1. Plant Materials and Pathogen Cultures

*Isatis indigotica* and *N. benthamiana* were grown in nutrient rich soil after pre-germination under controlled greenhouse conditions with 16/8 h light and dark intervals at 25 ± 3 °C. *Bacillus subtilis* 330-2 and *Xanthomonas oryzae* pv. oryzicola RH3 were maintained in the laboratory. *B. subtilis* WB800 was purchased from Takara Biomedical Technology (Dalian, China). *Ralstonia solanacearum* R21-5 was procured from the State Key Laboratory of Agricultural Microbiology, Huazhong Agricultural University, Wuhan, Hubei, China. *Xanthomonas oryzae* pv. oryzae XG-25 was procured from the Hubei Insect Resources Utilization and Sustainable Pest Management Key Laboratory, Huazhong Agricultural University, Wuhan, China. *Clavibacter michiganensis* subsp. The YCKYBI was procured from the Key Lab of Crop Disease Monitoring and Safety Control in Hubei Province, Huazhong Agricultural University, Wuhan, Hubei, China. All bacteria were maintained on Luria-Bertani (LB) media (tryptone, 10.0 g; yeast extract, 5.0 g; NaCl, 10.0 g per liter; pH 7.0). *Clavibacter fangii* 1.1999, purchased from the China General Microbiological Culture Collection Center, Beijing, China, was maintained on 0210 R agar media (tryptone, 10.0 g; yeast extract, 5.0 g; malt extract, 5.0 g; casamino acid, 5.0 g; beef extract, 2.0 g; glycerol, 2.0 g; Tween-80, 0.05 g; MgSO_4_·7H_2_O, 1.0 g; agar, 15.0 g, per liter; pH 7.2).

*Phytophthora capsici* (LT263), procured from the Key Laboratory of Crop Disease Monitoring and Safety Control, Huazhong Agricultural University, Wuhan, Hubei, China, was maintained on V8 media (V8 juice, 100 mL; CaCO_3_, 1 g; agar, 17 g per liter) at 24 ± 1 °C under dark conditions. *Rhizoctonia solani* (AG1-IA) and *B. cinerea* (B05.10) were maintained on potato dextrose agar (PDA) plates (dextrose, 200 g; agar, 17 g per liter; pH 7.0) at 28 ± 1 °C and 20 ± 1 °C, respectively. *Caenorhabditis elegans* N_2_ was maintained on nematode growth medium (NGM; tryptone, 2.5 g; NaCl, 3 g; agar, 20 g per liter; pH 7.5; after autoclaving, 1 mL of 1 M MgSO_4_, 1 mL of 1 M CaCl_2,_ 25 mL of 1 M KPO_4_ buffer and 1 mL of 5 mg/mL cholesterol were added and mixed well) at 24 ± 1 °C with *E. coli* OP_50_ as a food source. Further details are displayed in Table S1.

### 3.2. Isatis indigotica cDNA Library Construction

*Isatis indigotica* leaves were inoculated with *R. solani.* Samples were collected at different time intervals (6, 12, 24, 30, and 48 h), immediately frozen in liquid nitrogen, and saved at -80 °C. Total RNA was extracted by the Trizol method [28]. Next, mRNA was purified from total RNA using the PolyATtract^®^ mRNA isolation system (Promega, Madison, WI, USA). The cDNA library was created using the PrimeScript™ double-strand cDNA synthesis kit (Takara Biomedical Technology, Dalian, China) with specific oligo dT primers (containing an *Xba* I cleavage site), followed by linkage to three pairs of adaptors containing *Nde* I cleavage sites, as previously described [26]. Finally, cDNA products were transformed into *B. subtilis* WB800 cells. Individual colonies were picked and incubated at 37 °C overnight. Colony PCR was performed using pBE-S-F (5’-GTTATTTCGAGTCTCTACGG-3’) and pBE-S-R (5’-TAACCAAGCCTATGCCTACA-3’) primers to confirm the cDNA library quality and then saved at −80 °C.

### 3.3. Candidate Gene Screening and Confirmation

Initial screening and confirmation were performed as described in a previous study [25]. Briefly, an overnight culture was plated onto fresh LB plates supplemented with kanamycin (10 mg/L) and incubated at 37 °C to observe the phenotype. Strains showing cell lysis were selected for the next experiments. Plasmids were extracted from selected strains and transformed into *B. subtilis* again to reconfirm the gene function in cell lysis.

### 3.4. Scanning Electron Microscopy and Cytometric Analysis

Bacterial cell surface morphology was observed by SEM using previously described methods [29]. Briefly, *B. subtilis* as test indicator was grown in liquid LB supplemented with kanamycin (10 mg/L) and incubated at 37 °C for 36 h. Afterwards, bacterial cells were collected by centrifugation at 2500× *g* for 3 min. Next, three consecutive washing steps were performed with 20 mmol/L phosphate buffer saline (PBS; NaH_2_PO_4_·2H_2_O, 2.6 g; Na_2_HPO_4_·12H_2_O, 29 g; ddH_2_O 500 mL; pH 7.4). After washing, cells were resuspended in 2.5% glutaraldehyde and fixed for 2 h, followed by dehydration via an ethanol gradient, with 30% ethanol, 50% ethanol, 70% ethanol, 90% ethanol, and 100% ethanol. Dehydrated samples were then dried for 20 min before freeze-drying. Finally, cell samples were lyophilized and gold coated, and observed using a JEOL JSM-7001F scanning electron microscope (Toyama Prefecture, JAPAN).

To observe cell death events, as measured by PI staining, different levels of cell membrane damage of transgenic strains were analyzed on a FACSVerse machine (BD, Franklin Lake, NJ, USA) [30]. Confocal microscopy (Leica microsystems CMS GmbH TCS SP8; Leica, Germany) was also performed and cell samples were prepared as previously described [31]. Briefly, the *B. subtilis* strain was shaken at 37 °C for 36 h. Bacterial cells were harvested by centrifugation at 1000× *g* for 10 min and three consecutive washings were performed with 20 mM PBS buffer (pH 7.4), followed by resuspension in the same buffer at 1 × 10^9^ CFU/mL. Finally, the PI solution was diluted in the cell suspensions to achieve a final concentration of 40 mg/L, and the cells were stained for 30 min at 4 °C in the dark. After 30 min, the cells were washed and resuspended in the same volume of PBS buffer, and data were recorded and analyzed with Flowjo.7.6.1.Min (BD, Franklin Lake, NJ, USA). 

### 3.5. Analysis of Plasma Membrane Fluidity

To monitor plasma membrane fluidity, the DPH fluorescent probe was prepared and dissolved in tetrahydrofuran (THF) to a final concentration of 200 µM [32]. Then, *Bacillus* cells were collected and washed three times with 20 mmol/L PBS buffer (Na_2_HPO_4_, 0.3%; NaH_2_PO_4_, 0.6%, NaCl, 0.2%; (NH_4_)_2_SO_4_, 0.8%; pH 7.6). The final concentration of the cell pellets was set to OD_600_ 0.6. An amount of 2 µM DPH was dispersed in PBS and kept for 30 min at room temperature in the dark and, thereafter, the probe was removed by washing and resuspending in the same buffer. Fluorescence intensity was measured at an excitation wavelength of 365 nm and an emission wavelength of 425 nm.

### 3.6. Measurement of Membrane Potential

To monitor the membrane electrical potential (ΔΨ) of engineered bacteria, a fluorescent probe referred to as 3,3’-dipropylthiadicarbocyanine iodide (DiSC_3_(5); OR, USA) was used as previously described, with some modifications [33]. For the measurement of electrical potential gradients across the cytoplasmic membrane of integral cells, excitation wavelengths were set to 622 nm and emission wavelengths were set to 670 nm. Cells were washed and resuspended in 20 mmol/L potassium HEPES buffer (HEPES, 20 mM; NaCl, 153 mM; KCl, 5 mM; glucose, 5 mM; pH 7.4). DiSC_3_(5) was dispersed in HEPES buffer at a final concentration of 5 µM, and after 3 min of incubation, cells were washed three times with HEPES buffer, followed by fluorescence intensity measurement with a spectrofluorometer (Shimadzu RF- 5301 PC, Kyoto, Japan).

### 3.7. Detection of Cell Membrane Integrity

To measure cell membrane integrity, we monitored nucleic acid outflow using an Eppendorf Bio Photometer (Hamburg, Germany) [34]. Overnight grown bacteria were inoculated into 50 mL liquid LB supplemented with kanamycin (10 mg/L) and shaken at 37 °C. Cell samples were filtrated with 0.22 µm filters to remove the bacteria completely. The absorbance of the supernatant was measured at 260 nm every 2 h.

### 3.8. Expression of Crude Proteins

Extracellular peptides were precipitated using the ammonium sulfate precipitation method with slight modifications [35]. *Bacillus subtilis* was grown in 200 mL liquid LB supplemented with kanamycin (10 mg/L) and incubated at 180 r/min, 37 °C for 72 h. Supernatants were collected by centrifugation at 10,000× *g*, 4 °C for 20 min. Extracellular peptides were precipitated by adding a saturated ammonium sulfate solution to a final concentration of 50–70% and stirring continuously on ice for at least 20 min, followed by storage at 4 °C for 12 h and then centrifugation to pellet the peptide. The precipitated peptides were dissolved in PBS buffer (pH 7.0) at a concentration of 25 mM and dialyzed in the same PBS buffer for 24 h at 4 °C. The insoluble debris were discarded by centrifugation using the same conditions previously used.

### 3.9. Antimicrobial Activity and Thermal Stability Assays

Antibacterial activity assays were performed using the previously described disk diffusion method [36]. Briefly, indicator bacteria (10^8^ CFU/mL) were mixed with semisolid NA media and poured over previously prepared NA plates. Then, 5 mm diameter filter paper disks were placed on the agar plates and 20 µL of secreted peptides (1000 mg/L) were added to each filter paper. Thereafter, the Petri dishes were incubated at the different temperatures required for the different indicator bacteria for 12 h. Antibacterial activities were confirmed by measuring the zones of inhibition. For thermal stability tests, the peptides were heated at 50, 75, and 100 °C for 30 min before use.

Inhibition assays for *B. cinerea* was performed as described previously, with slight modifications [37]. Test peptides were prepared as above. Approximately 200 µL of peptides were mixed with 4 mL of semisolid PDA media and poured onto previously prepared PDA plates to a final concentration of 128 mg/L. Then, the plates were seeded with 5 × 5 mm mycelial plugs taken from the periphery of three-day-old colonies of *B. cinerea* (B05.10), followed by incubation at 20 °C. The *B. subtilis* WB800-e strain was used as a control. Data were recorded at 48 hpi. Percent inhibition was calculated according to the following equation: Percent inhibition (%) = [(diameter of control − diameter of treatment)/(diameter of control − diameter of pathogen disk)] × 100%. 

### 3.10. Generation of His-Tag Fusion Peptides

To purify the IiR515 and IiR915 peptides, His-tag fusion genes were constructed. To add restriction sites at corresponding positions in the coding sequences of *IiR515* and *IiR915*, specific primers were designed (Table S3). Transformation steps were followed based on previously described methods [38]. After transformation, the phenotypes of the cells and their antimicrobial activities were assessed.

### 3.11. Purification of Extracellular Peptides

IiR515 and IiR915 were purified using nickel column affinity chromatography and their antimicrobial activities were confirmed. The fermentation process was carried out as described previously [35]. Briefly, supernatants were collected by centrifugation at 10,000× *g* and 4 °C for 20 min. Thereafter, target peptides were captured using a nickel column at 4 °C, eluted with 600 nm imidazole, and then concentrated with a 3.5 kDa ultrafiltration tube.

### 3.12. Tris-Tricine SDS-PAGE and Western Blotting

The purified peptide samples were mixed with 2X Tricine-SDS-PAGE Loading Buffer (CWBIO) at a 1:1 proportion, heated at 100 °C for 3–5 min, and centrifuged at 13,000× *g* for 2 min to remove precipitated impurities. Peptides were separated on a 16.5% gel and electrophoresed by sodium dodecyl sulphate polyacrylamide gel electrophoresis (SDS-PAGE) with a Tris-Tricine buffer system as described previously [39]. Then, immunoblots were performed with mouse anti-His-tag monoclonal antibody, goat anti-mouse IgG (H+L), HRP, and ECL detection reagents. The molecular weights were predicted by comparing with color pre-dyed ultra-low peptide molecular weight marker (Well Biotech, Chungju-si, Korea) [40].

### 3.13. Resistance Determination Test

Resistance tests were performed with detached *N. benthamiana* leaves using a previous method [41]. Uniformly sized leaves were collected and soaked in pure peptides (100 μg/mL), which were pretreated with 0.05% Silwet L-77, for 3 sec. *Bacillus subtilis* WB800-e was used as a control. Then, the leaves were inoculated with 5 × 5 mm disks of *P. capsici* mycelium and incubated at 25 ± 3 °C with high humidity in the dark. Data were recorded at 48 hpi to calculate the percent inhibition according to the following equation: [(control lesion diameter − treatment lesion diameter)/(control lesion diameter − pathogen disk diameter)] × 100%.

The *IiR515* and *IiR915* genes were ligated into the pTRV_2_Ex vector and transformed into Agrobacterium tumefaciens EHA105; they were named *TRV_2_-IiR515* and *TRV_2_-IiR915*, respectively. Leaves from uniformly sized *N. benthamiana* plants were selected for infiltration (one side of the leaf) with *TRV_2_-IiR515* and *TRV_2_-IiR915,* followed by incubation at 25 ± 3 °C with high humidity. The pTRV_2_ empty vector was used as a control. At 48 hpi, infiltrated *N. benthamiana* leaves were collected and inoculated with *B. cinerea* (other side of the leaf). Data and images were recorded at 48 hpi. Percent inhibition was calculated according to the same equation described above.

### 3.14. Anti-Soil-Borne Bacteria Assay

Anti-soil-borne bacteria assays were performed in simulated environments. Sterilized pots were filled with approximately 100 g of sterilized soil. Then, a 15 mL amount of fermentation broth was evenly mixed with the prepared soil. *Bacillus subtilis* WB800-e was used as a control. After 48 h, soil-borne pathogens were prepared at the same concentration (10^8^ to 10^9^ CFU/mL), and pretreated soil was inoculated with a total of 15 mL of pathogen culture. Soil samples were collected at different time intervals (0, 24, 48, and 72 h) and 10 g of soil was mixed with 90 mL sterilized double distilled water. The mixture was shaken at 180 r/min, 28 °C for 90 min; this was the stock solution for gradient dilutions to obtain an optimum concentration (10^−5^ dilution). A total of 100 µL of each dilution was taken and spread onto LB plates, followed by incubation at 28 °C. The data were recorded at 24 hpi.

### 3.15. Hemolytic Activity of IiR515 and IiR915 Peptides

Hemolytic activity of the IiR515 and IiR915 peptides was assessed using sheep erythrocytes according to previously described methods [42]. Briefly, fresh sheep blood was centrifuged at 600× *g* for 10 min to collect the erythrocytes, then washed three times with PBS buffer (0.2 M, pH 7.2) and resuspended in same solution (1% *v*/*v*). Different concentrations of pure peptides were diluted with precooled PBS buffer, mixed with isopycnic erythrocyte suspensions in a 96-well cell culture plate and incubated at 37 °C for 1 h. Thereafter, the cell culture plates were centrifuged at 600× *g* for 10 min and 70 µL of the supernatants from each well were collected into a new 96-well cell culture plate. Data were recorded at 540 nm using a microplate spectrophotometer (xMarK BIO RAD, California USA). An erythrocyte suspension treated with 1% Triton X-100 was used as a positive control, whereas a suspension incubated with only PBS buffer was used as a negative control. The hemolytic activity percentage was calculated by the following equation: hemolysis (%) = [(OD_540_ peptides − OD_540_ buffer)/(OD_540_ Triton X-100 − OD_540_ buffer)] × 100%.

## 4. Discussion

There has been increasing interest in the isolation of AMPs in the drug-screening research field recently. In previous studies, scientists have successfully used cDNA libraries for the investigation of protein–protein interactions [43] and the identification of antimicrobial peptides from different organisms [44]. *Bacillus subtilis* as a host cell facilitates soluble and secretory protein expression, and it is particularly effective for studying the activities of peptides and proteins [45].

For the isolation of novel AMPs, we established a new, sensitive, and high-throughput strategy based on the damaging or killing effects of peptides against *B. subtilis* host cells [25]. A drawback of this strategy is that strong AMPs will kill *B. subtilis* cells too rapidly to detect the clones, and therefore, only AMPs with weak killing effects are preferred. However, *B. subtilis* has a good secretory system, which may reduce the toxicity of strong AMPs. Additionally, during the first few hours (12 h), the concentration of strong AMPs is possibly not high enough to kill the cells, making the selection of strong AMPs feasible. In our study, part of the *B. subtilis* system selected AMPs that did not actually show a strong effect on *B. subtilis* cells; however, the selected AMPs did show strong antimicrobial activity against other pathogens. Although the selection of AMPs with weak killing effects is preferred, the *B. subtilis* screening system has the ability to select for cell membrane-interrupting antimicrobial peptides that inhibit a broad range of pathogens. This strategy was used for the screening of AMPs from the Chinese herbal plant, *I. indigotica*, which was previously reported for its high antimicrobial activities [46] and other health-related benefits [47]. In this study, we identified 45 different candidates from *I. indigotica* with the potential to induce autolysis of *Bacillus* cells. Out of the 45 candidates, two different peptides, IiR515 and IiR915, that exhibited strong antimicrobial activities were selected for further study. These two peptides were considered to be novel because they had no homology in NCBI BLAST (National Center for Biotechnology Information and Basic Local Alignment Search Tool) searches or the antimicrobial peptide database (APD) (Figure S4). 

Antimicrobial peptides interact with pathogens and enhance their membrane permeability as a killing mechanism [48]; destroying the cell membrane is an efficient way to kill microorganisms [49]. In our study, SEM observations and confocal microscopy images showed cytomembrane damage, including shrinkage, distortion, pore formation, and ruptures. Cytometric analysis of *IiR515*- and *IiR915*-transformed *Bacillus* cells showed the same results and were supported by previous studies [50]. Propidium iodide can penetrate broken cell membranes and cause the majority of cells to emit red fluorescence. Cytomembrane breakage always accompanies intracellular component outflow, such as nucleic acids, which can be detected in the extracellular environment. Cell membrane damages also correlate with membrane fluidity and changes in electrical potential, as our results show. We treated the genomes of different pathogens with these peptides, but they did not cause DNA degradation. Based on our findings, we assume the possible mechanism of the IiR515 and IiR915 peptides was to interrupt or rupture the cell membrane. 

Antimicrobial activity tests revealed that the products of our cDNA library contain many antimicrobial peptides, which can directly interact with pathogens. *Bacillus subtilis* clones exhibiting antimicrobial activities were identified on the basis of the proteins that accumulated inside the cells and caused cell autolysis. Correct guidance by signal peptides can ensure successful secretion of different proteins [51]. *Bacillus subtilis* 168 is the most popular host for the expression of heterogenous proteins, and it is a biocontrol agent [52] that can secrete many extracellular proteases to degrade extracellular proteins. In contrast, the strain used in this study was *B. subtilis* WB800, which has mutations in eight key extracellular proteases to reduce the degradation of extracellular proteins [53]. There are several methods that exist for cDNA library construction, which have a significant influence on the experimental results. Many of the methods require PCR amplification to increase the abundance of cDNAs. However, there is a major drawback with these methods, in that a large number of repeated genes are selected in subsequent steps; however, in our present method, PCR amplification is not required to increase the abundance of the cDNA library.

Antimicrobial peptides have been reported to have antimicrobial activities against a diverse range of microorganisms. Both the IiR515 and IiR915 peptides were consistent with previously reported AMPs because of their significant inhibitory effects against Gram-positive and Gram-negative bacteria. Minimum inhibitory concentration for recombinant pBD142 were 100 µg/mL and 80 µg/mL against *Escherichia coli* and *Staphylococcus aureus,* respectively. Similarly, the MIC for AMP CAP-1 from *Pseudomonas* sp. ranged from 30 to 550 µg/mL against a wide range of pathogens [36,54]. Minimum inhibitory concentration for our peptides revealed that they were similar to the previously reported AMPs. However, on the other hand, the inhibition activity of our peptides was not observed as strong as for vancomycin [55]. *Phytophthora capsici* is a pathogenic oomycete which is hemi-biotrophic and has a broad range of hosts. It can infect most of the Cucurbitaceae and Solanaceae crops [56]. Disks containing *P. capsici* were used to inoculate *N. benthamiana* leaves for pathogenicity analysis by observation of necrotic lesions [57]. Our results demonstrated that IiR515 and IiR915 enhanced the resistance of *N. benthamiana* towards *P. capsici*. These two peptides were then ligated into the pTRV_2_Ex (viral vector) vector, and we observed that they had the potential to improve the resistance of *N. benthamiana* towards *B. cinerea*. Previous studies have shown that the use of viral vectors can produce a huge quantity of AMPs in plant systems to efficiently reduce pathogen attack [58], as was observed in our study.

Antimicrobial peptides exhibit damaging effects against some organisms, but their safety in animals and humans is still an open question [59]. As previously reported, some AMPs have shown low hemolytic activity against mammalian blood cells [1]. In our study, low hemolytic activities were observed at extremely high peptide concentrations. Additionally, different bioassays using *C. elegans* were performed to assess the toxicity of IiR515 and IiR915 against nematodes [60]. Although the IiR515 and IiR915 peptides have effects on the food tropism of *C. elegans*, these two peptides did not show any killing effect on *C. elegans*. Furthermore, there were no significant differences in the number of offspring or the body length (Supplementary toxicity assay and Figure S5). Therefore, we speculated that these two peptides obtained from *I. indigotica* are relatively safe for animal cells and may also be safe for humans. These results indicate the possibility for the application of AMPs to animals and humans in the future.

From the present research, we conclude that the peptides derived from *I. indigotica* are novel in their functions against different pathogens. These peptides have low molecular weight, a broad antibacterial spectrum, and a good stability against a wide range of temperatures. Furthermore, these peptides also have the ability to destroy the bacterial cell membrane and cell wall structures. On the basis of these distinctive features, future research can be designed for the further characterization of these peptides, followed by the generation of resistance in different crops, as they have already been revealed as potential candidates against pathogens. We hope that, in the future, these peptides can be used as potential raw materials for drug discovery. 

## Supplementary Materials

The following are available online at https://www.mdpi.com/2218-273X/10/1/30/s1, Figure S1: Quality assessment of total RNA, mRNA, and cDNA, Figure S2: Agarose gel to show cDNA inserts, Figure S3: Antibacterial activity of precipitated peptides against different bacteria performed using an agar diffusion assay, Figure S4: Sequence alignment analysis of IiR515 and IiR915, Figure S5: Peptide secretion assay of IiR515- and IiR915-transformed *B. subtilis* strains against *C. elegans*; Table S1: List of strains and vectors used in this study, Table S2: Inhibition of candidate peptides against different microorganisms, Table S3: Primers used to construct His6-IiR915 peptide, Table S4: MICs of IiR515 and IiR915 against different microorganisms, Table S5: Hemolytic activity of IiR515 and IiR915 against sheep blood cells. Supplementary minimum inhibitory concentration (MIC) assay and toxicity assay.
