# Supplementary material for: Cell Membrane-Interrupting Antimicrobial Peptides from Isatis indigotica Fortune Isolated by a Bacillus subtilis Expression System
Source: Biomolecules. 2019 Dec 24;10(1):30. doi: 10.3390/biom10010030 (PMC7023251; doi:10.3390/biom10010030)
Supplement: Supplementary file 1 [file biomolecules-10-00030-s001.pdf]

Supplementary Materials

Cell membrane interrupting antimicrobial peptides from *Isatis indigotica* Fort isolated by a *Bacillus subtilis* expression system

Jia Wu, Hafiz Muhammad Khalid Abbas, Jiale Li, Yuan Yuan, Yunjun Liu, Guoying Wang & Wubei Dong

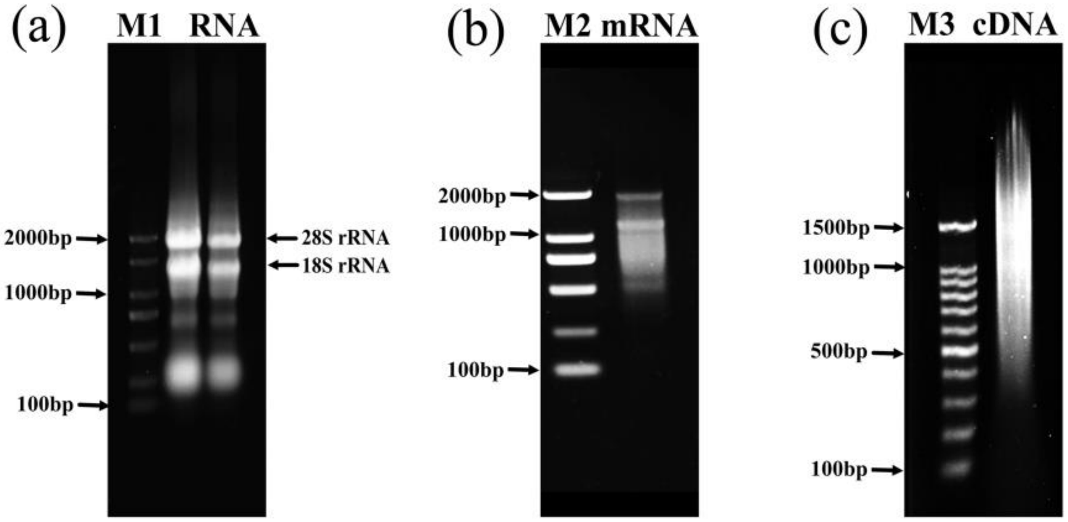

Figure S1 Quality assessment of total RNA, mRNA and cDNA

(a) Total RNA was extracted using Trizol method, (b) mRNA was purified from total RNA by PolyATtract® mRNA isolation systems (Promega), and (c) double strand cDNA was synthesized from mRNA using PrimeScript™ double strand cDNA synthesis kit (TaKaRa).

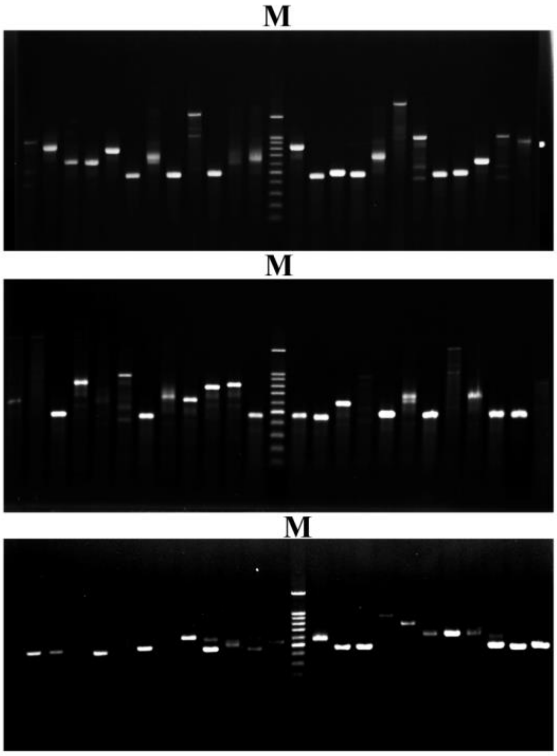

Figure S2 Agarose gel to show cDNA inserts

M: 100 bp DNA marker.

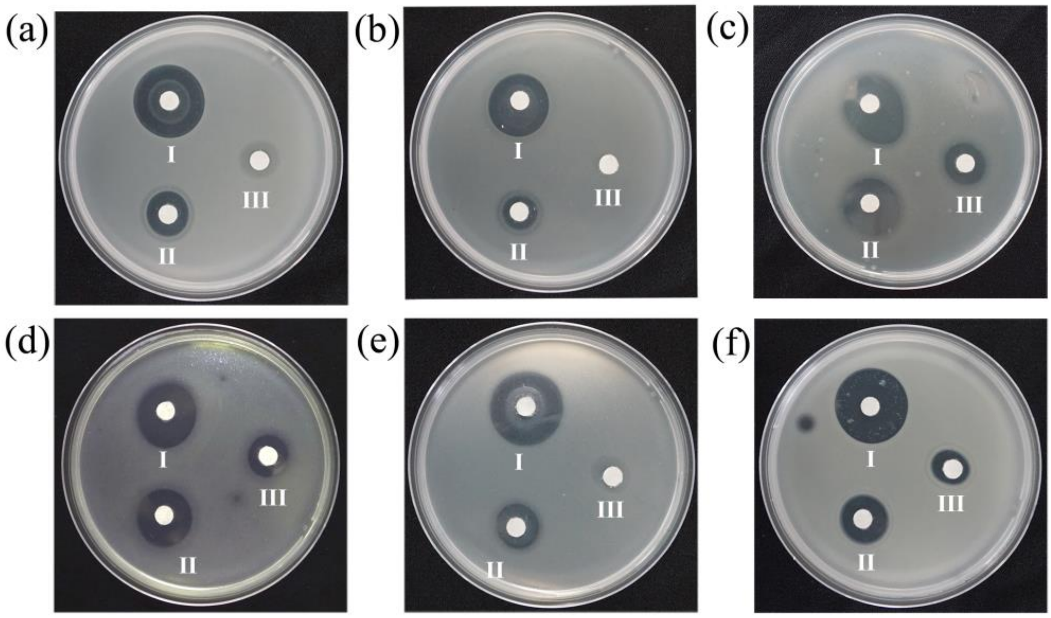

Figure S3 Antibacterial activity of precipitated peptides against different bacteria performed using an agar diffusion assay

Bacteria were preincubated to the mid logarithmic phase in LB media (LB) and adjusted the concentration (10^8 CFU/mL). About 400 µL of bacterial culture was mixed with 4 mL semi solid NA medium and poured, then, added antibacterial peptides. (a) *C. fangii* 1.1999 as indicator, (b) *C. michiganensis* subsp YCKYBI as indicator, (c) *B. subtilis* 330-2 as indicator, (d) *X. oryzae* pv. oryzae XG-25 as indicator, (e) *X. oryzae* pv. oryzicola RH3 as indicator, (f) *R. solanacearum* R21-5 as indicator. The clear inhibition zoon of *IiR*515 (I), *IiR*915 (II), *B. subtilis* WB800-e **(**III**),** were observed obvious. Each assay was replicated individual thrice

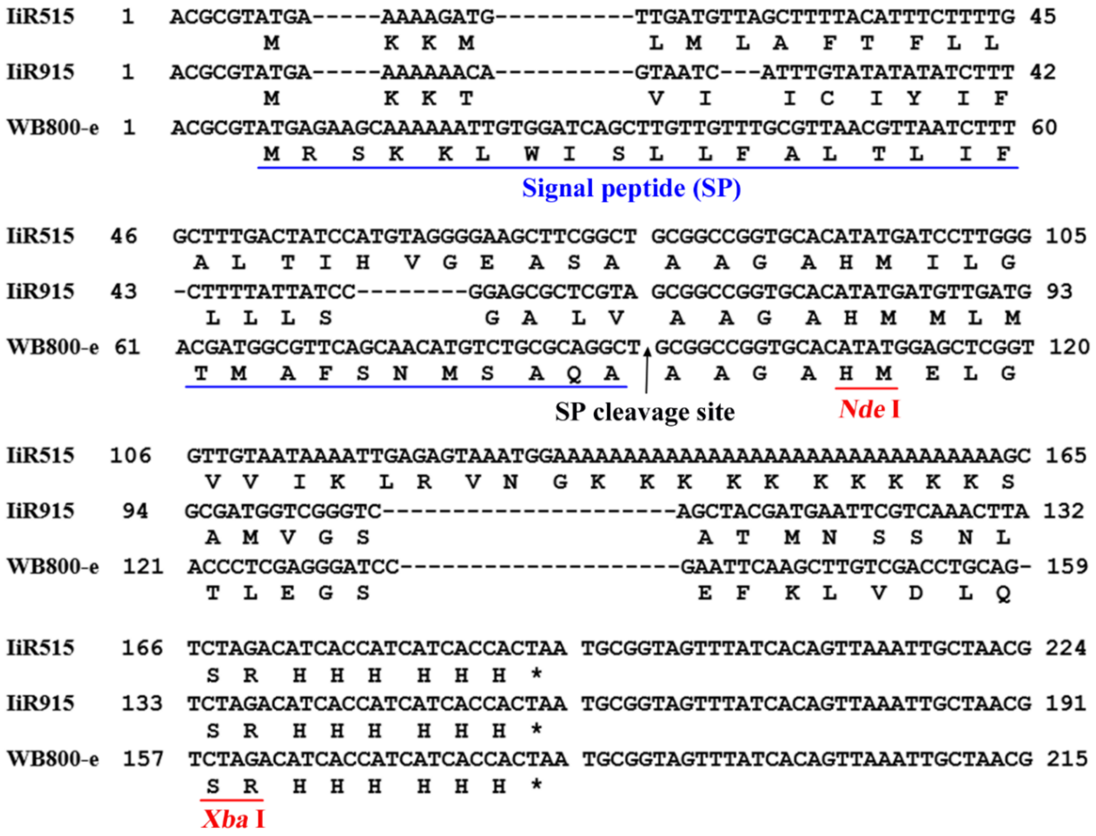

Figure S4 Sequence alignment analysis of *IiR515* and *IiR915*

Note: Stop codon indicated by asterisks.

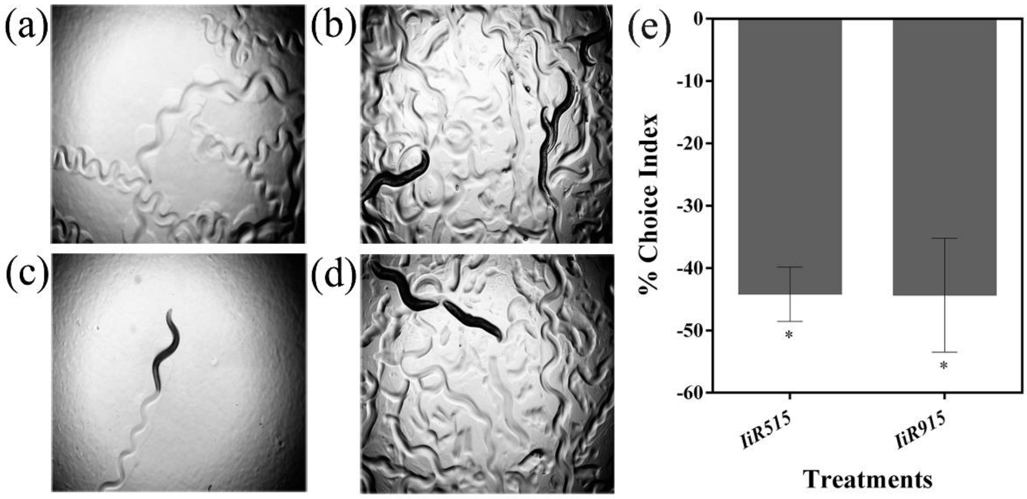

Figure S5 Peptide secretion assay of IiR515- and IiR915-transformed *B. subtilis* strains against *C. elegans*

Illustration of worms and their movements under light microscope in response to (a) IiR515, (b) WB800-e, (c) IiR915 and (d) WB800-e. (e) Choice index for *C. elegans*. Data are the mean values from three individual experiments. Vertical bars represent the SD. For significance analysis *t*-tests were performed: * $p<0.05$.

Table S1 List of strains and vectors used in this study

Materials	Description	Reference
**Bacterial Strains**		
Bacillus subtilis WB800	*trpC2 nprE aprE epr bpr mpr nprB vpr wprA;* Hygr	[1,2]
B. subtilis 330-2	New *Bacillus* strain isolated by our lab	[3]
B. subtilis WB800-e	WB800 transformed with unmodified expression vector	This study
*B. subtilis*168	*trpC2*	[4]
B. cereus 905	Wild type strain isolated from wheat rhizosphere	[5]
Bacillus sp. K1	A positive *Bacillus* strain	This study
Escherichia coli OP_{50}	Av uracil requiring mutant of *E. coli*	[6]
E. coli HST08	High-efficiency competent cells	This study
Ralstonia solanacearum R21-5	Soil-borne plant pathogenic bacteria	This study
C. michiganensis subsp. YCKYB1	An aerobic non-sporulating gram-positive plant pathogenic bacteria	This study
C. fangii 1.1999	Wheat blight pathogen	CMCC
X. oryzae pv. oryzae XG-25	A seed - borne and transmitted pathogen, can cause a serious bacterial leaf blight of rice	This study
X. oryzae pv. oryzicola RH3	A seed - borne and transmitted pathogen, can cause a serious bacterial leaf blight of rice	[7]
X. campestris pv. holcicola	Non-pathogenic species on wild rapeseed	CMCC

Pectobacterium carotovorum subsp. carotovorum	Pathogenic species on maize	CMCC
**plasmids**		
pBE-S	*B. subtilis-E. coli* shuttle vector	TaKaRa
$pTRV_2Ex$	A *Tobacco rattle virus* (TRV)-based VIGS vector	[8]
**Fungi**		
Phytophthora capsici LT263	Plant pathogenic oomycete that cause blight	[9,10]
Rhizoctonia solani AG1-IA	A soil-borne fungus pathogen causing foliar blight and rot disease	[11]
Botrytis cinerea B05.10	The causal agent of the grey mold disease	[12]
Fusarium sp.	Cause stalk and ear rot diseases on maize, and seedling blight on maize, wheat and barley	This study
Alternaria sp.	A pathogenic and saprophytic bacterium in the environment	[13]
**Nematode**		
C. elegans N2	A model host for biological experiments applying	[14]

Table S2 Inhibition of candidate peptides against different microorganisms

	Test genes	*WB800*	*IiR205*	*IiR505*	*IiR515*	*IiR576*	*IiR595*
**Fungi**	***Fusarium* sp.**	-	ND	ND	-	ND	ND
	Botrytis cinerea	-	ND	ND	+	ND	ND
	P. capsici	-	ND	ND	-	ND	ND
	R. solani	-	ND	ND	-	ND	ND
	***Alternaria* sp.**	-	ND	ND	-	ND	ND
**Gram-negative bacteria**	***X. oryzae* pv. oryzae**	3.000 ± 1.528	ND	ND	14.33 ± 2.333	ND	ND
	***X. oryzae* pv. oryzicola**	0.6667 ± 0.333	ND	ND	13.67 ± 1.856	ND	ND
	***X. campestris* pv. holcicola**	3.250 ± 0.479	ND	ND	4.750 ± 0.250	ND	ND
	***P. carotovorum* subsp. carotovorum**	-	ND	ND	-	ND	ND
	R. solanacearum	2.333 ± 1.856	13.75 ± 1.109	15.75 ± 1.315	14.00 ± 1.528	11.75 ± 0.479	12.00 ± 0.577
**Gram-positive bacteria**	***C. fangii***	2.667 ± 1.667	ND	ND	13.33 ± 1.764	ND	ND
	C. michiganensis	0.667 ± 0.333	14.67 ± 1.764	13.75 ± 1.436	13.33 ± 1.667	8.000 ± 0.577	11.00 ± 0.577
	***Bacillus* sp. K1**	4.333 ± 0.882	10.33 ± 1.202	11.33 ± 0.333	11.67 ± 1.333	ND	ND
	***B. subtilis* 330-2**	5.333 ± 0.333	14.00 ± 1.528	15.33 ± 0.333	14.67 ± 2.603	9.333 ± 0.333	7.667 ± 0.333
	***B. cereus* 905**	ND	9.333 ± 1.453	9.333 ± 0.882	15.00 ± 0.577	ND	6.500 ± 0.645
	***B. subtilis* 168**	ND	9.500 ± 1.500	13.50 ± 2.500	13.33 ± 0.333	10.00 ± 0.408	ND
	***B. subtilis* WB800-e**	ND	7.750 ± 0.479	8.000 ± 0.408	8.000 ± 1.528	5.250 ± 0.479	7.833 ± 0.441

IiR699	*IiR773*	*IiR898*	*IiR915*	*IiRFR*	*IiR1268*	*IiR1301*	*IiR1413*	*IiR1510*
ND	ND	ND	-	-	ND	ND	ND	ND
ND	ND	ND	+	+	-	ND	ND	ND
ND	ND	ND	-	-	-	ND	ND	ND
ND	ND	ND	-	-	-	ND	ND	ND
ND	ND	ND	-	-	-	ND	ND	ND
ND	ND	ND	9.000 ± 1.528	9.600 ± 1.208	ND	ND	ND	10.00 ± 0.408
ND	ND	ND	8.667 ± 1.202	8.000 ± 1.225	ND	ND	ND	8.000 ± 0.577
ND	ND	ND	5.000 ± 0.408	5.500 ± 0.289	ND	ND	ND	ND
ND	ND	ND	-	-	ND	ND	ND	ND
12.67 ± 1.202	12.25 ± 0.478	ND	11.00 ± 1.732	8.100 ± 0.822	13.00 ± 1.528	10.33 ± 0.333	10.00 ± 0.408	12.50 ± 0.645
ND	ND	ND	8.667 ± 0.667	10.40 ± 0.510	9.000 ± 0.447	ND	ND	ND
9.667 ± 0.882	6.000 ± 0.577	6.667 ± 0.333	8.000 ± 1.155	10.17 ± 0.441	13.33 ± 0.667	5.667 ± 0.333	11.50 ± 0.289	-
10.00 ± 0.577	6.000 ± 0.577	7.250 ± 0.479	6.333 ± 0.667	7.333 ± 0.882	9.667 ± 0.333	6.667 ± 0.882	ND	11.67 ± 0.333
11.67 ± 1.202	10.00 ± 1.000	9.000 ± 1.528	11.67 ± 0.882	12.00 ± 0.816	13.67 ± 0.882	11.00 ± 0.577	ND	15.75 ± 0.629
9.250 ± 1.109	6.000 ± 0.408	9.250 ± 0.479	13.00 ± 0.577	8.000 ± 0.577	10.50 ± 0.645	9.500 ± 0.289	ND	8.667 ± 1.202
5.000 ± 3.000	8.000 ± 0.577	7.000 ± 0.577	11.67 ± 0.333	8.000 ± 1.000	9.000 ± 2.000	9.667 ± 1.453	8.667 ± 0.882	11.00 ± 0.577
8.400 ± 0.245	8.667 ± 0.333	10.00 ± 2.082	10.50 ± 1.088	7.667 ± 1.202	12.67 ± 0.333	10.00 ± 1.528	ND	-

ND: Not detected; -: No inhibition; Data presented here, showing mean value of inhibition diameter (mm) and standard deviation from three individual experiments.

Table S3 Primers used to construct His6-IiR915 peptide

Primers	5' to 3'
915F	GGGTTTCATATGATGTTGATGGCGATGGTCGGGTCAGCTACGATG
915R	TACGTCTAGATAAGTTTGACGAATTCATCGTAGCTGACCCGAC

Table S4 MICs of IiR515 and IiR915 against different microorganisms

Microorganisms	MIC (µg/mL)	
	IiR515	IiR915
C. fangii	45 (10.6 µM)	80 (24.5 µM)
C. michiganensis	70 (16.5 µM)	50 (15.3 µM)
X. oryzae	45 (10.6 µM)	50 (15.3 µM)
R. solanacearum	50 (11.8 µM)	80 (24.5 µM)

Note: MIC: Minimal inhibitory concentration. Data represent the mean values of three replicates. µM: Micromolar. The MIC data are also provided in the unit of µM in parenthesis.

Table S5 Hemolytic activities of IiR515 and IiR915 against sheep blood cells

**Treatments**	**Percentage hemolysis at different concentrations (µg/mL)**				
	**0**	**125**	**250**	**500**	**1000**
IiR515	0.00 ± 0.00	0.60 ± 0.60	1.83 ± 1.83	5.00 ± 3.21	5.30 ± 0.70
IiR915	0.00 ± 0.00	0.00 ± 0.00	0.40 ± 0.60	0.60 ± 0.60	7.50 ± 4.27
PBS	0.00 ± 0.00	0.00 ± 0.00	0.00 ± 0.00	0.00 ± 0.00	0.00 ± 0.00
Triton X-100	100.00 ± 0.00	100.00 ± 0.00	100.00 ± 0.00	100.00 ± 0.00	100.00 ± 0.00

Supplementary methods

Minimum inhibitory concentration (MIC) assay

The MIC was determined as previously described [15]. Briefly, indicator bacteria were grown in LB media, and set to final OD of 0.02–0.05. Different concentrations of pure peptides were diluted with precooled PBS buffer, mixed with cell culture in a 96-well cell culture plate and incubated at 37°C for 24 h. Thereafter, data were recorded at 600 nm using a microplate spectrophotometer (xMarK BIO RAD). Cell culture mixed with PBS was used as positive control, and PBS buffer mixed with LB media was used as a negative control.

Supplementary toxicity assay

Toxicity assay of IiR515 and IiR915 against *C. elegans*

For toxicity assays, IiR515 and IiR915 were used against *C. elegans* according to previous methods [16]. Pure peptides were spotted onto NGM plates at a final concentration of 100 ng/µL, and after 24 h of incubation, *E. coli* OP_{50} was spotted onto the same place and the plates were again incubated for 12 h. L_4 stage *C. elegans* was placed at the center of the plates at an equal distance from both bacterial lawns. The number of worms was counted after 12 h. Choice index was determined according to following formula: Choice index (%) = (the number of worms counted on the test lawn - the number of worms counted on the OP_{50} lawn) / the total number of worms * 100%. The toxicities of IiR515 and IiR915 on *C. elegans* were tested. The IiR515 and IiR915 peptides have effects on the food tropism of *C. elegans*. However, these two peptides did not show any killing effect on *C. elegans*. Furthermore, the number of offspring and the body length were not influenced by these two peptides.

References

1. Koichiro Murashima; Chyi-Liang Chen; Akihiko Kosugi; Yutaka Tamaru; Roy H. Doi; Wong, S.-L. Heterologous production of *Clostridium cellulovorans eng* B, using protease-deficient *Bacillus subtilis*, and preparation of active recombinant cellulosomes. *Journal of bacteriology* **2002**, *184*, 76-81.
2. Zhang, X.Z.; Cui, Z.L.; Hong, Q.; Li, S.P. High-level expression and secretion of methyl parathion hydrolase in *Bacillus subtilis* WB800. *Applied and Environmental Microbiology* **2005**, *71*, 4101-4103.
3. Ahmad, Z.; Wu, J.; Chen, L.; Dong, W. Isolated *Bacillus subtilis* strain 330-2 and its antagonistic genes identified by the removing PCR. *Scientific Reports* **2017**, *7*, 1-13.
4. Zeigler, D.R.; Pragai, Z.; Rodriguez, S.; Chevreux, B.; Muffler, A.; Albert, T.; Bai, R.; Wyss, M.; Perkins, J.B. The origins of 168, W23, and other *Bacillus subtili*s legacy strains. *J Bacteriol* **2008**, *190*, 6983-6995.
5. Wang, Y.; Wang, H.; Yang, C.H.; Wang, Q.; Mei, R. Two distinct manganese-containing superoxide dismutase genes in *Bacillus cereus*: Their physiological characterizations and roles in surviving in wheat rhizosphere. *FEMS Microbiology Letters* **2007**, *272*, 206-213.
6. Brenner, S. The genetics of *Caenorhabditis elegans Genetics* **1974**, *77*, 71-94.
7. Yang, W.; Xu, X.; Li, Y.; Wang, Y.; Li, M.; Wang, Y.; Ding, X.; Chu, Z. Rutin-mediated priming of plant resistance to three bacterial pathogens initiating the early sa signal pathway. *PloS ONE* **2016**, *11*, 1-15.
8. Choi, D.S.; Hwang, B.K. Proteomics and functional analyses of pepper abscisicacid–responsive 1 (ABR1), which is involved in cell death and defense signaling. *The Plant Cell* **2011**, *23*, 823-842.
9. Wang, Y.; Bouwmeester, K.; van de Mortel, J.E.; Shan, W.; Govers, F. A novel Arabidopsis-oomycete pathosystem: differential interactions with *Phytophthora capsici* reveal a role for camalexin, indole glucosinolates and salicylic acid in defence. *Plant, Cell & Environment* **2013**, *36*, 1192-1203.
10. Qiao, Y.; Shi, J.; Zhai, Y.; Hou, Y.; Ma, W. *Phytophthora* effector targets a novel component of small RNA pathway in plants to promote infection. *PNAS* **2015**, *112*, 5850-5855.

11. Ciampi, M.B.; Meyer, M.C.; Costa, M.J.; Zala, M.; McDonald, B.A.; Ceresini, P.C. Genetic structure of populations of *Rhizoctonia solani* anastomosis group-1 IA from soybean in Brazil. *Phytopathology* **2008**, *98*, 932--941.
12. Dean, R.; A L Van Kan, J.; Pretorius, Z.; Hammond-Kosack, K.; Di Pietro, A.; Spanu, P.; Rudd, J.; Dickman, M.; Kahmann, R.; Ellis, J., *et al.* The top 10 fungal pathogens in molecular plant pathology. *Molecular Plant Pathology* **2012**, *13*, 414-430.
13. Ramires, F.A.; Masiello, M.; Somma, S.; Villani, A.; Susca, A.; Logrieco, A.F.; Luz, C.; Meca, G.; Moretti, A. Phylogeny and Mycotoxin Characterization of Alternaria Species Isolated from Wheat Grown in Tuscany, Italy. *Toxins* **2018**, *10*, 1-15.
14. Gray, J.C.; Cutter, A.D. Mainstreaming *Caenorhabditis elegans* in experimental evolution. *Proceedings. Biological Sciences* **2014**, *281*, 1-10.
15. Barns, K.J.; Weisshaar, J.C. Real-time attack of LL-37 on single *Bacillus subtilis* cells. *Biochim Biophys Acta* **2013**, *1828*, 1511-1520.
16. Lewenza, S.; Charron-Mazenod, L.; Giroux, L.; Zamponi, A.D. Feeding behaviour of *Caenorhabditis elegans* is an indicator of *Pseudomonas aeruginosa* PAO1 virulence. *PeerJ* **2014**, *2*, 1-19.
